# The relationship between COVID-19, depressive disorder, and anxiety: a bidirectional Mendelian randomization study

**DOI:** 10.3389/fpsyt.2023.1257553

**Published:** 2023-10-19

**Authors:** Liang Zihao, Song Jinyun, Gu Shuanglin, Chen Xiuzhen, Li Yonglin, Zhao Hongyu

**Affiliations:** ^1^Clinical Research Center, The Second Hospital of Nanjing, Nanjing University of Chinese Medicine, Nanjing, China; ^2^The First Affiliated Hospital of Nanjing Medical University, Nanjing Medical University, Nanjing, China

**Keywords:** COVID-19, anxiety, depression, Mendelian randomization, GWAS

## Abstract

**Background:**

Previous clinical studies have found that negative mental states such as depression and anxiety are closely related to COVID-19 infection. We used Mendelian randomization (MR) to explore the relationship between depression, anxiety, and COVID-19 infection.

**Methods:**

Our data were based on publicly available GWAS databases. The COVID-19 samples were obtained from the COVID-19 Host Genetics Initiative (HGI). The depression samples were obtained from the Psychiatric Genomics Consortium (PGC). The anxiety samples were derived from the Finngen database. We used inverse-variance weighting (IVW) as the primary analysis method, with weighted median, MR Egger, and multivariate MRI adjustment.

**Results:**

There was no causal effect of different COVID-19 infection statuses on depression and anxiety as determined by MR analysis. In addition, in the reverse MR analysis, we found a significant causal effect of anxiety on severe symptoms after COVID-19 infection. The results of the MR Egger regression, weighted median, and weighted mode methods were consistent with the IVW method. Based on sensitivity analyses, horizontal pleiotropy was unlikely to influence the final results.

**Conclusion:**

Our findings indicate that anxiety is a risk factor for severe symptoms following COVID-19 infection. However, the mechanism of interaction between the two needs further investigation.

## 1. Introduction

From 2019 to 2022, COVID-19 spread worldwide, causing severe public health issues on a global scale. It is an infectious disease caused by the Severe Acute Respiratory Syndrome Coronavirus 2 (SARS-CoV-2), with an estimated 2.75 billion persons at risk of infection (1). Despite the end of the pandemic, many patients have been found to have acute SARS-CoV-2 sequelae, also known as long COVID or post-COVID-19 syndrome (2). The World Health Organization (WHO) defines it as a condition in which individuals who have been diagnosed or may have been infected with SARS-CoV-2 in the past have persistent symptoms within 3 months of onset that persist for at least 2 months and cannot be explained by an alternative diagnosis (3).

The main symptoms of COVID-19 sequelae include shortness of breath, cognitive dysfunction, fatigue, anxiety, and depression (4). Compared to the latest WHO incidence rates for common mental health disorders, the incidence of depression in patients with COVID-19 was three times higher (15.97%) than in the general population; The prevalence of anxiety disorders is four times higher than in the general population (15.15% higher than in the general population) (5).

Depression is a prevalent mental illness that affects many individuals. In clinical practice, the most common symptoms are a depressed mood, a lack of interest, and impaired cognitive function (6). According to the most recent data, there are approximately 264 million patients worldwide (7). Major depressive disorder (MDD) can even lead to suicide and death. Depression has arisen as a risk factor for numerous illnesses. Many studies have shown that there is a significant increase in the incidence of depression in people infected with COVID-19 (8). Clinical studies have suggested that anxiety and depression are risk factors for COVID-19 infection and will lead to a longer recovery period after COVID-19 infection (9, 10). Due to the negative effects of social isolation and disruptions in health services on people's mental health and wellbeing, researchers believe that the increase in depression following COVID-19 infection is likely to be comparable to the increase following other previous pandemics, such as SARS (severe acute respiratory syndrome) and MERS (Middle East respiratory syndrome coronavirus) (4, 11).

Genetic factors significantly influence the susceptibility to and the severity of a wide range of infectious diseases and psychiatric disorders. Several recent studies have found the same genetic factor linking psychiatric disorders and infectious diseases. The presence of numerous SNP sites on the HLA gene associated with psychiatric disorders and mutations in these sites may affect the immune response to foreign antigens, which may account for the increased incidence of infections and inflammation in patients with schizophrenia and bipolar disorder and their parents (12). A large Danish genomic study identified 90 SNPs associated with mental disorders and susceptibility, most notably rs6447952 (13). Chen analyzed GWAS data from populations with psychiatric disorders and COVID-19 infections utilizing polygenic risk scores and found that genetic susceptibility to psychiatric disorders correlated with the risk of COVID-19 and severe COVID-19 (14).

Mendelian randomization (MR) is an epidemiological research technique that uses genetic variants as instrumental variables to infer the causality of a risk factor because it employs genetic variants as instrumental variables (15, 16). Mendelian randomization is independent of environmental factors and self-selected lifestyle choices (17). When the sample size is adequate, and the genetic variant is not associated with potential confounders, the quasi-random assignment of that variant outside of the exposure level ought to produce groups with nearly identical characteristics on average. MR analysis is now widely used to analyze causal relationships between diseases and risk factors, e.g., between gut microbes and disease, between two different diseases, and between metabolites and disease (18). Therefore, we used Mendelian randomization to determine whether COVID-19 as the exposure and depression/anxiety as the outcome were directly causally related.

## 2. Materials and methods

### 2.1. Design of experiment

**Figure 1:**
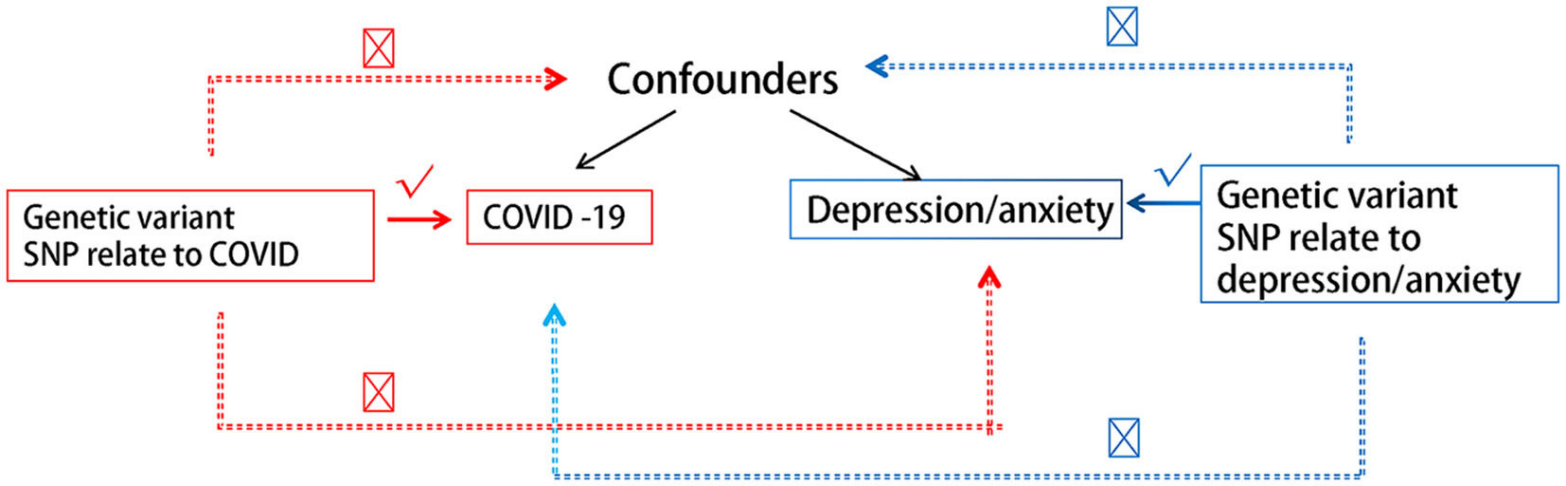
Description of this bidirectional Mendelian randomization experiment.

We hereby briefly describe the design of the bidirectional MR between COVID-19 and depression/anxiety. Using pooled data from genome-wide association studies (GWAS), we performed two MR analyses to examine bidirectional associations between various COVID-19 statuses and depression/anxiety. Reverse MR analyses used depression/anxiety as exposure and distinct COVID-19 statuses as outcomes. Figure 1 depicts the fundamental hypotheses of MR. Using three guiding principles, this study hypothesizes the following (17, 19, 20):

(1) There is a substantial association between genetic variation and exposure.
(2) Genetic mutations are unrelated to other confounding variables.
(3) Only exposure is associated with genetic variation and outcome.

Based on summary statistics available to the public, this research did not require ethical approval.

### 2.2. Data sources

We attempted to perform MR analysis using the COVID-19 GWAS data. The COVID-19 dataset was obtained from the COVID-19 Host Genetics Initiative (HGI). GWAS provided us with the association between COVID-19 and COVID-19 genetic associations of phenotypes. The GWAS yielded three phenotypes: (1) COVID-19 patients and the general population (38,984 cases and 1,644,784 controls); (2) hospitalized COVID-19 patients and the general population (3,159 cases and 7,206 controls); and (3) patients with severe respiratory confirmed COVID-19 and the general population (5,101 cases and 1,383,241 controls) (21). Depression data were obtained from the Psychiatric Genomics Consortium (PGC), which currently contains 807,553 individuals (246,363 cases and 561,190 controls) (22, 23). The data on patients with anxiety disorders were obtained from the Finngen database (40,191 cases and 277,526 controls). To exclude the influence of ethnicity, we chose a cohort of European populations. Details and sources of the data are given in Table 1.

**Table 1:** Specific information and sources of GWAS data.

**Phenotype**	**Trait contains**	**ID**	**Source**
COVID-19	Very severe respiratory confirmed COVID-19	A2_ALL_eur_leave_23andme	https://www.COVID19hg.org
	Hospitalized COVID-19	B2_ALL_eur_leave_23andme	
	COVID-19	C2_ALL_eur_leave_23andme	
Depression	Major depression	mdd2019edinburgh	https://pgc.unc.edu/for-researchers
Anxiety	Anxiety disorders (more control exclusions)	Psychiatric endpoints from Katri Räikkönen	https://r9.finngen.fi/

### 2.3. Screen of instrumental variable (IV) for MR analysis

In order to obtain appropriate instrumental variables from different GWAS data, we first selected genome-wide significant SNPs (*p* < 5 × 10^−8^) (24). To ensure linkage disequilibrium of instrumental variables, we chose kb = 10,000, r^2^ < 0.001 as a condition. Finally, in order to evaluate the tool strength, we made sure the *F* > 10 ones were used as instrumental variables (25, 26). We then harmonized the exposure and outcome datasets to obtain genetic instrument effects on the outcome and to remove palindromic SNPs.

### 2.4. Statistical analysis

Using a random-effects inverse variance weighting (IVW) method, we estimated the bidirectional causality between COVID-19 status and depression/anxiety. The IVW method presupposes that all MR assumptions are legitimate. However, IV influenced the results through other pathways, indicating that horizontal pleiotropic effects may exist and that estimates of IVW causality may be biased. Therefore, we conducted sensitivity analyses utilizing the MR Egger and weighted median methodologies, allowing us to estimate causality accurately even in the presence of invalid SNPs.

As MR relies on the three central IV assumptions of the primary analysis (Figure 1), we hereby describe the methods used to evaluate or demonstrate the validity of these assumptions. The correlation hypothesis calculates r^2^, which indicates the proportion of the exposure variable's variation that can be explained by genetic variation. We calculated the *f*-statistics to evaluate the instrumental intensity of the relationship between IV and interest exposure risk. *F* represents weak instrumental vigor. MR Egger regression intercepts and their respective 95% confidence intervals (CIs) were utilized to examine the extent to which directional pleiotropy, which precludes limiting assumptions, leads to bias in arbitrary estimates. Moreover, horizontal pleiotropy was evaluated using the Mendelian randomized pleiotropy residuals and outliers (MR-PRESSO) global test, and the outlier SNPs were excluded using the MR-PRESSO outlier test. Additionally, after removing the peripheral IV, we examined whether there was a statistically significant difference between the new IV and the previous one. Using Cochran's Q statistic and funnel diagrams, we also examined the IVW and MR Egger methods for heterogeneity. Then, various sensitivity analyses (such as leave-one-out and individual SNP analyses) were conducted to determine whether individual SNPs affected primary causality. Using odds ratios (OR) and 95% confidence intervals (CIs), we estimated causality for binary outcomes. We presented causal estimates, *p*-values, and their standard errors for both binary and continuous outcomes. Each *p*-value is bilateral. All analyses were conducted utilizing the R (version 4.3.0, www.r-project.org) TwoSampleMR and Mendelian randomization packages.

## 3. Results

### 3.1. Screening of genetic tools

We obtained 51 SNPs as instrumental variables in depression, 54 in anxiety, 29 in very severe respiratory confirmed COVID-19, 33 in COVID-19 hospitalization COVID-19 SNPs, and 15 SNPs in COVID-19 infection, which met the generally accepted genome-wide significance threshold (*p* < 5 × 10 ^−8^, r^2^ < 0.001, kb = 10,000) for exposure. However, anxiety was adjusted to a significance threshold of *p* < 5 × 10 ^−6^ because only a few SNPs were acquired. Detailed data are provided in Supplementary material 1.

### 3.2. Causal effects of COVID-19 infection on anxiety and depression

**Figure 2:**
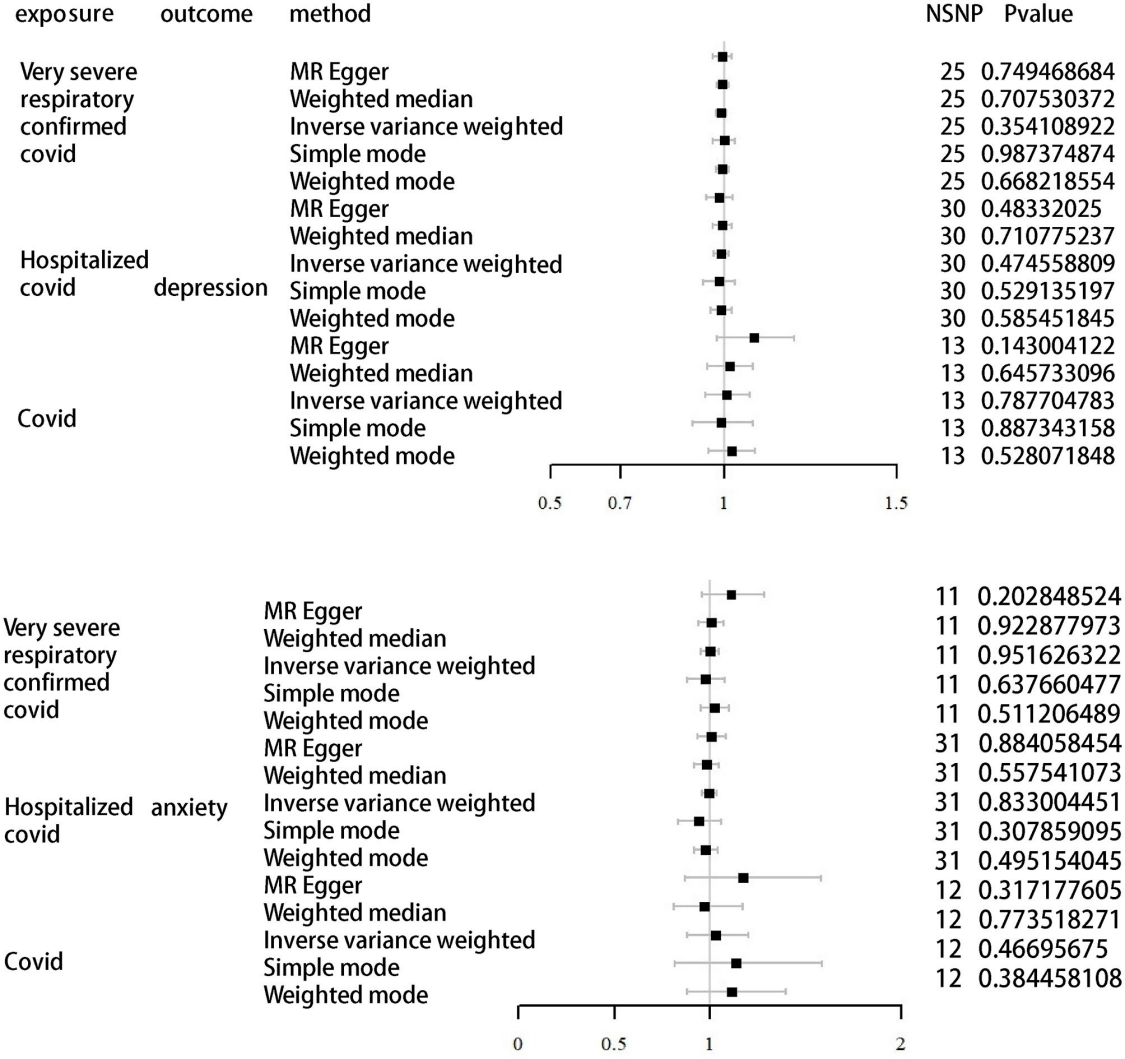
MR analysis of depression and anxiety as outcomes using different infections of COVID-19 as exposure.

As shown in Tables 2, 3 and Figure 2, the IVW results indicated no significant correlation between the genetically predicted COVID-19 infection profiles and depression and anxiety. For example, very severe respiratory confirmed COVID-19 showed no significant association with anxiety (OR, 0.99; 95% CI, 0.95–1.04; *P* = 0.95) and depression (OR, 0.99; 95% CI, 0.97–1.01; *P* = 0.35). Similarly, COVID-19 requiring hospitalization exhibited no significant relation to anxiety (OR, 0.83; 95% CI, 0.96–1.04; *P* = 0.83) and depression (OR, 0.99; 95% CI, 0.97–1.01; *P* = 0.47), while COVID-19 infection also presented with no significant association with anxiety (OR, 0.83; 95% CI, 0.96–1.04; *P* = 0.83) and depression (OR, 1.01; 95% CI, 0.95–1.07; *P* = 0.79). Further analyses using MR Egger regression, weighted median, and weighted mode methods continued to show no causal association between different COVID-19 infection statuses and depression/anxiety. Detailed information can be found in Supplementary material 2.

**Table 2:** Association of different COVID-19 statuses with depression in MR analysis.

**Exposures**	**Outcome**	**Method**	**NSNP**	**B**	**SE**	**PVAL**
Very severe respiratory confirmed COVID-19	Depression	MR Egger	25	−0.004750305	0.014698014	0.749468684
		Weighted median	25	−0.003462482	0.009228979	0.707530372
		Inverse variance weighted	25	−0.007485112	0.008077615	0.354108922
		Simple mode	25	0.000260369	0.016283651	0.987374874
		Weighted mode	25	−0.004118848	0.009492068	0.668218554
Hospitalized COVID-19		MR Egger	30	−0.014133196	0.019894181	0.48332025
		Weighted median	30	−0.005353026	0.014435836	0.710775237
		Inverse variance weighted	30	−0.007569588	0.010585632	0.474558809
		Simple mode	30	−0.015398662	0.024174515	0.529135197
		Weighted mode	30	−0.00839524	0.015219947	0.585451845
COVID-19		MR Egger	13	0.082818581	0.052502673	0.143004122
		Weighted median	13	0.015392815	0.033484634	0.645733096
		Inverse variance weighted	13	0.008645516	0.032104582	0.787704783
		Simple mode	13	−0.006519406	0.045052035	0.887343158
		Weighted mode	13	0.021868586	0.033654926	0.528071848

**Table 3:** Association of different COVID-19 statuses with anxiety in MR analysis.

**Exposures**	**Outcome**	**Method**	**NSNP**	**B**	**SE**	**PVAL**
Very severe respiratory confirmed COVID-19	Anxiety	MR Egger	11	0.10405562	0.075761299	0.202848524
		Weighted median	11	0.00313751	0.032409234	0.922877973
		Inverse variance weighted	11	−0.001468431	0.024205732	0.951626322
		Simple mode	11	−0.025030973	0.051538129	0.637660477
		Weighted mode	11	0.024661144	0.036202126	0.511206489
Hospitalized COVID-19		MR Egger	31	0.005487875	0.037303033	0.884058454
		Weighted median	31	−0.019340422	0.032976124	0.557541073
		Inverse variance weighted	31	−0.004411899	0.020924363	0.833004451
		Simple mode	31	−0.06280267	0.060541055	0.307859095
		Weighted mode	31	−0.022371918	0.032397031	0.495154045
COVID-19		MR Egger	12	0.160125115	0.152084869	0.317177605
		Weighted median	12	−0.027206087	0.094539137	0.773518271
		Inverse variance weighted	12	0.028543326	0.080379876	0.722510653
		Simple mode	12	0.128634762	0.170710516	0.46695675
		Weighted mode	12	0.106043511	0.117072928	0.384458108

### 3.3. Causal effects of anxiety and depression on COVID-19 infection

**Figure 3:**
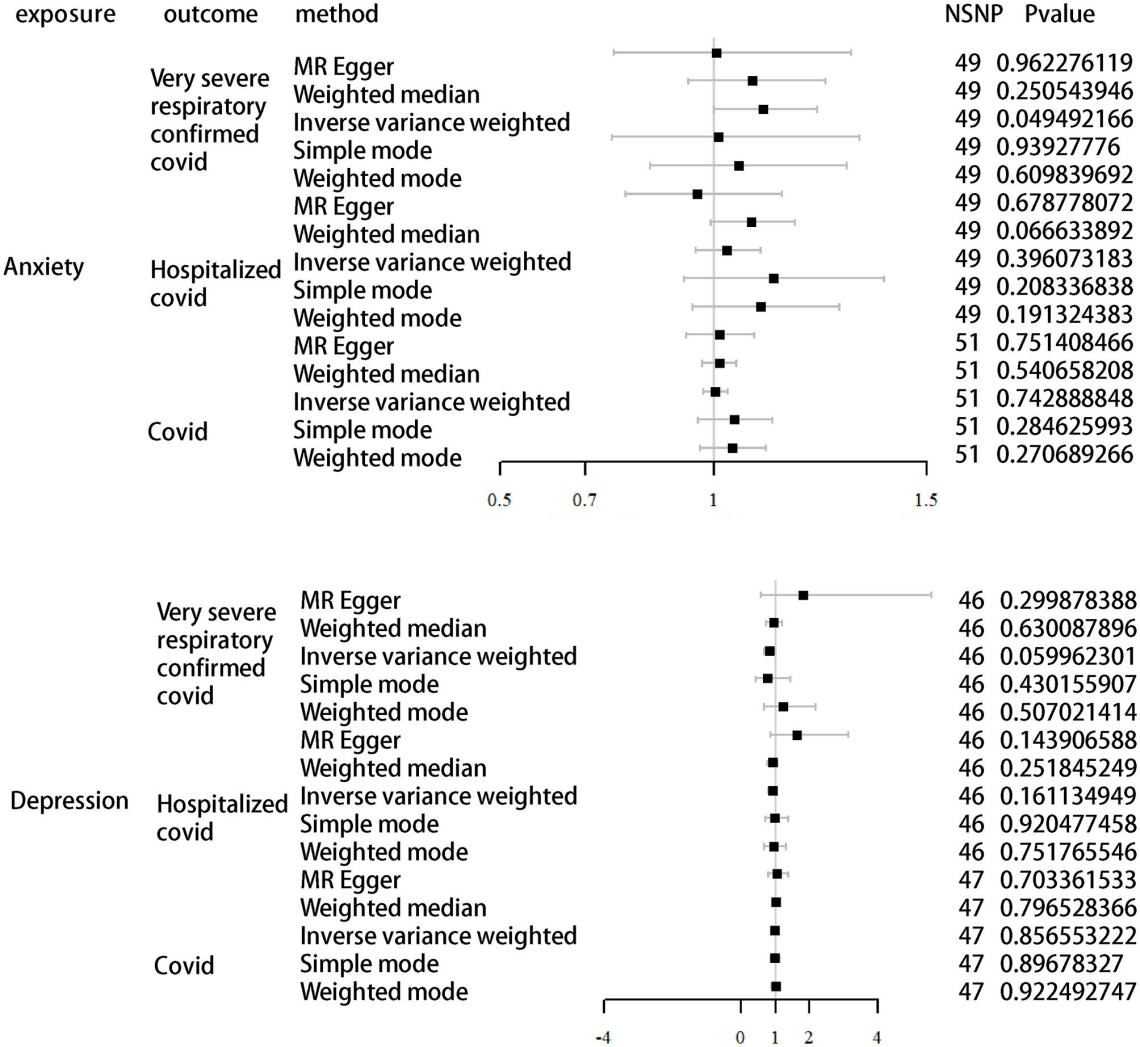
MR analysis of different infections of COVID-19 as outcomes using depression and anxiety as exposure.

We further validated the relationship between different COVID-19 infection statuses and depression/anxiety using directional MR. As shown in Table 4 and Figure 3, the results differ from previous ones in that we found a possible correlation between anxiety and severe illness after COVID-19 infection. We set depression and anxiety as the exposure factors and different COVID-19 infection statuses as outcomes. Depression for very severe respiratory confirmed COVID-19 (OR, 0.82; 95% CI, 0.67–1.01; *P* = 0.06), depression for hospitalized COVID-19 (OR, 0.91; 95% CI, 0.81–1.04; *P* = 0.16), and depression for COVID-19 (OR, 1.00; 95% CI, 0.95–1.05; *P* = 0.86). Anxiety for very severe respiratory confirmed COVID-19 (OR, 1.12; 95% CI, 1.00–1.24; *P* = 0.05), anxiety for hospitalized COVID-19 (OR, 1.03; 95% CI, 0.96–1.11; *P* = 0.40), and anxiety for COVID-19 (OR, 1.00; 95% CI, 0.98–1.03; *P* = 0.74).

**Table 4:** Association of anxiety/depression with different COVID-19 statuses in MR analysis.

**Exposures**	**Outcome**	**Method**	**NSNP**	**B**	**SE**	**PVAL**
Depression	Very severe respiratory confirmed COVID-19	MR Egger	46	0.598459	0.570471	0.299878
		Weighted median	46	−0.06259	0.129955	0.630088
		Inverse variance weighted	46	−0.19771	0.105107	0.059962
		Simple mode	46	−0.24836	0.311976	0.430156
		Weighted mode	46	0.200381	0.2996	0.507021
Anxiety		MR Egger	49	0.006646308	0.139774233	0.962276119
		Weighted median	49	0.086616086	0.075381972	0.250543946
		Inverse variance weighted	49	0.109121703	0.055551695	0.049492166
		Simple mode	49	0.011127597	0.145311095	0.93927776
		Weighted mode	49	0.056740439	0.110460996	0.609839692
Depression	Hospitalized COVID-19	MR Egger	46	0.495696	0.333149	0.143907
		Weighted median	46	−0.09427	0.082273	0.251845
		Inverse variance weighted	46	−0.08681	0.061954	0.161135
		Simple mode	46	−0.01787	0.178002	0.920477
		Weighted mode	46	−0.05624	0.176718	0.751766
Anxiety		MR Egger	49	−0.040552251	0.097312214	0.678778072
		Weighted median	49	0.08425579	0.045937605	0.066633892
		Inverse variance weighted	49	0.031754872	0.037417872	0.396073183
		Simple mode	49	0.133054135	0.104330645	0.208336838
		Weighted mode	49	0.104582907	0.078908792	0.191324383
Depression	COVID-19	MR Egger	47	0.05368	0.140077	0.703362
		Weighted median	47	0.008824	0.034224	0.796528
		Inverse variance weighted	47	−0.0046	0.025446	0.856553
		Simple mode	47	−0.0099	0.075865	0.896783
		Weighted mode	47	0.006712	0.068608	0.922493
Anxiety		MR Egger	51	0.012916928	0.040546914	0.751408466
		Weighted median	51	0.012513017	0.020452191	0.540658208
		Inverse variance weighted	51	0.004884418	0.014890149	0.742888848
		Simple mode	51	0.046435184	0.042932531	0.284625993
		Weighted mode	51	0.042550178	0.038202593	0.270689266

## 4. Discussion

In this study, we explored whether there is a causal relationship between COVID-19 infection and anxiety/depression. As described in the results, we found that there does not appear to be a highly significant causal link between COVID-19 infection and anxiety/depression. Only anxiety disorders were causally associated with severe reactions after COVID-19 infection, and anxiety disorders may be a risk factor for severe illness after COVID-19 infection.

Previous observational clinical studies have found that more than 50% of the infected patients have depression or anxiety-like symptoms after COVID-19 infection, and anxiety and depression are also the typical symptoms of COVID-19 sequelae considered by the WHO (27, 28). In addition, researchers from the United Kingdom discovered that those infected with COVID-19 who were hospitalized were 49% more likely to be diagnosed with depression, anxiety, or a mental condition than those infected but not hospitalized (29, 30). This finding is consistent with what was discovered in the Nordic countries, where patients who had been hospitalized for more than 7 days had a significantly higher risk of depression and anxiety than those who had not been hospitalized (31). According to the results of our investigation, the presence of COVID-19 infection, the severity of the COVID-19 disease, and hospitalization for COVID-19, all appeared to have no direct causal effect on the development of depression or anxiety. As a consequence of the findings of other investigations that have been published, a number of theories have been developed. One possible explanation for the depressive and anxious symptoms exhibited by patients is that these conditions are at least partially caused by the patients' social environment (32). This may include social isolation and high levels of stress. The main sources of stress during the COVID-19 pandemic were fear of infection, frustration, boredom, lack of supplies, and economic loss (33). Fear of infection and occupational stress (increased work pressure on healthcare workers during a pandemic, increased unemployment due to changes in the socio-economic environment, increased uncertainty about the future due to a pandemic and thus academic stress, etc.) were the main causes of increased stress during a pandemic (34–37). Excessive stress is one of the major causes of mental disorders such as depression and anxiety (38).

During the COVID-19 pandemic, many individuals have been required to maintain a safe distance from one another to prevent the spread of COVID-19, resulting in social isolation (39). It is believed that social isolation causes sleep disturbances (40). Insomnia and sleep disorders are recognized as major risk factors for the development of depression and anxiety (41). Regarding social isolation-induced insomnia as a mechanism leading to melancholy and anxiety, scientists believe social isolation results in hypothalamic–pituitary–adrenal (HPA) axis dysfunction (42). The disorder of the HPA axis induces hyperexcitation and sleeplessness in the human body (43). The prevalence of insomnia symptoms (36.7%) and insomnia disorders (17.4%) during the COVID-19 pandemic was approximately double the prevalence reported during non-pandemic periods, with higher rates in Brazil, Canada, the United Kingdom, and the United States, where depression and anxiety rates have also increased (44).

Intensive research on inflammation and psychiatry suggests that immune system perturbations triggered by infection may specifically promote psychopathology, increasing the psychological stress of living with a potentially fatal illness and stress-related inflammation (45). Interactions between the innate and adaptive immune system and neurotransmitters underlie mood disorders, psychosis, and anxiety disorders. Similar results have been observed in the past for similar pandemics. Some researchers believe that this is due to the virus infecting the neural tissue, resulting in the latter's inflammatory response. It has been demonstrated that coronavirus has neurophilic properties and can infect brain tissues. Additionally, COVID-19 has been detected in the cerebrospinal fluid (46–49).

Mental factors such as depression and anxiety are important risk factors for many diseases, such as cardiovascular diseases, digestive tract diseases, and susceptibility to viruses (50, 51). Our study found a genetic causal link between anxiety disorders and symptom severity following COVID-19 infection. This is consistent with the current clinical studies that have found anxiety or depression to be a risk factor for COVID-19 infection. Patients who are depressed or anxious are not only more likely to be infected with COVID-19 than the general population but also appear to have more severe symptoms after infection (52). In addition, some studies have found that patients with depression and anxiety have a longer recovery period after COVID-19 infection, which may be related to immune dysregulation caused by the HPA system (53). The detection of serum cortisol in infected patients revealed that COVID patients had higher cortisol levels and that elevated cortisol levels were positively correlated with mortality after COVID-19 infection (54, 55). Cortisol plays a key role in the development of depression and anxiety. Is it the elevated cortisol caused by depression and anxiety that makes patients more susceptible to COVID-19 infection and more severe symptoms? Whether the increased cortisol caused by COVID-19 may lead to subsequent increases in depression and anxiety requires further research.

The very interesting finding in our study is that anxiety seems to be a risk factor for developing severe illness after a COVID-19 infection. However, depression does not seem to increase the risk of developing severe illness after COVID-19 infection. Anxiety disorders are often accompanied by autonomic arousal compared to depression (56). In addition, clinical studies have found that depressed patients have lower catecholamine levels than the normal population, while anxious patients have higher catecholamine levels than the normal population (57, 58). During the inflammatory response, catecholamine concentrations are elevated, which in turn exacerbate inflammation by promoting the secretion of pro-inflammatory cytokines such as IL-6, IL-1β, and tumor necrosis factor, especially in myeloid cells (59). This inflammatory response may be exacerbated by the higher catecholamine levels in patients with anxiety disorders themselves, leading to a more severe inflammatory response after COVID-19 infection than in normally infected individuals, with a higher chance of causing a severe reaction.

However, the results regarding the causal relationship between anxiety and the risk of severe illness after COVID-19 infection do not seem to be strong. Meanwhile, a large number of clinical studies have shown that a poor psychological state prior to COVID-19 infection is a key factor in triggering severe COVID-19 disease after infection (60). Chen et al. also analyzed GWAS data from the UK Biobank. They found that depression and anxiety were more likely to result in severe and fatal COVID-19 infections (14). We therefore consider our results to be plausible.

This study also has some limitations. First, the data we selected were from a European population lacking generalizability. We also needed more specific raw data for subgroup analyses. In future studies, we will increase the sample size, expand the population to include different ethnic groups, and collect appropriate subgroup information for more in-depth analyses.

## 5. Conclusion

We used a much broader population sequence than in previous studies. Our study identified anxiety disorders as a risk factor for the development of severe symptoms following COVID-19 infection. Patients with anxiety disorders are more likely to have severe symptoms after COVID-19 infection than the general population. Although this association does not appear to be strong, given that anxiety disorders are risk factors for a wide range of diseases, we should pay more attention to people with anxiety disorders during future infectious disease pandemics.

## Data availability statement

The original contributions presented in the study are included in the article/Supplementary material, further inquiries can be directed to the corresponding author.

## Author contributions

LZ and ZH conceived the idea for this study. LZ, GS, and SJ obtained genetic data and performed data analysis. LZ and SJ were responsible for revising the article. CX and LY explained the results of the data analysis. ZH wrote the article. All authors approved the submitted version.

## Supplementary material

Supplementary Material 1 Instrumental variables SNPs.

Supplementary Material 2 MR results between COVID and anxiety/depression.

## References

[1] Ceban F Ling S Lui LMW Lee Y Gill H Teopiz KM. Fatigue and cognitive impairment in Post-COVID-19 syndrome: a systematic review and meta-analysis. Brain Behav Immun. (2022) 101:93–135. 10.1016/j.bbi.2021.12.020 34973396 PMC8715665

[2] Yong SJ. Long COVID or post-COVID-19 syndrome: putative pathophysiology, risk factors, and treatments. Infect Dis (Lond). (2021) 53:737–54. 10.1080/23744235.2021.1924397 34024217 PMC8146298

[3] Montani D Savale L Noel N Meyrignac O Colle R Gasnier M. Post-COVID-19 syndrome. Bull Acad Natl Med. (2023) 207:812–20. 10.1016/j.banm.2023.01.029 37292432 PMC10126882

[4] Ramakrishnan RK Kashour T Hamid Q Halwani R Tleyjeh IM. Unraveling the mystery surrounding post-acute sequelae of COVID-19. Front Immunol. (2021) 12:686029. 10.3389/fimmu.2021.686029 34276671 PMC8278217

[5] Cenat JM Blais-Rochette C Kokou-Kpolou CK Noorishad PG Mukunzi JN McIntee SE. Prevalence of symptoms of depression, anxiety, insomnia, posttraumatic stress disorder, and psychological distress among populations affected by the COVID-19 pandemic: A systematic review and meta-analysis. Psychiatry Res. (2021) 295:113599. 10.1016/j.psychres.2020.113599 33285346 PMC7689353

[6] Monroe SM Harkness KL. Major Depression and Its Recurrences: Life Course Matters. Annu Rev Clin Psychol. (2022) 18:329–57. 10.1146/annurev-clinpsy-072220-021440 35216520

[7] Nomura S Sakamoto H Glenn S Tsugawa Y Abe SK Rahman MM. Population health and regional variations of disease burden in Japan, 1990-2015: a systematic subnational analysis for the Global Burden of Disease Study 2015. Lancet. (2017) 390:1521–38. 10.1016/S0140-6736(17)31544-1 28734670 PMC5613077

[8] Robinson E Sutin AR Daly M Jones A. A systematic review and meta-analysis of longitudinal cohort studies comparing mental health before versus during the COVID-19 pandemic in 2020. J Affect Disord. (2022) 296:567–76. 10.1016/j.jad.2021.09.098 34600966 PMC8578001

[9] Blackett JW Wainberg M Elkind MSV Freedberg DE. Potential long coronavirus disease 2019 gastrointestinal symptoms 6 months after coronavirus infection are associated with mental health symptoms. Gastroenterology. (2022) 162:648–650 e642. 10.1053/j.gastro.2021.10.040 34728186 PMC8556689

[10] Garjani A Middleton RM Nicholas R Evangelou N. Recovery from COVID-19 in multiple sclerosis: a prospective and longitudinal cohort study of the united kingdom multiple sclerosis register. Neurol Neuroimmunol Neuroinflamm. (2022) 9:1. 10.1212/NXI.0000000000001118 34848503 PMC8631790

[11] Jeong H Yim HW Song YJ Ki M Min JA Cho J. Mental health status of people isolated due to Middle East Respiratory Syndrome. Epidemiol Health. (2016) 38:e2016048. 10.4178/epih.e2016048 28196409 PMC5177805

[12] Avramopoulos D Pearce BD McGrath J Wolyniec P Wang R Eckart N. Infection and inflammation in schizophrenia and bipolar disorder: a genome wide study for interactions with genetic variation. PLoS ONE. (2015) 10:e0116696. 10.1371/journal.pone.0116696 25781172 PMC4363491

[13] Nudel R Wang Y Appadurai V Schork AJ Buil A Agerbo E. A large-scale genomic investigation of susceptibility to infection and its association with mental disorders in the Danish population. Transl Psychiatry. (2019) 9:283. 10.1038/s41398-019-0622-3 31712607 PMC6848113

[14] Chen W Zeng Y Suo C Yang H Chen Y Hou C. Genetic predispositions to psychiatric disorders and the risk of COVID-19. BMC Med. (2022) 20:314. 10.1186/s12916-022-02520-z 35999565 PMC9397166

[15] Sekula P Del Greco MF Pattaro C Kottgen A. Mendelian randomization as an approach to assess causality using observational data. J Am Soc Nephrol. (2016) 27:3253–65. 10.1681/ASN.2016010098 27486138 PMC5084898

[16] Bowden J Holmes MV. Meta-analysis Mendelian randomization: a review. Res Synth Methods. (2019) 10:486–96. 10.1002/jrsm.1346 30861319 PMC6973275

[17] Davey Smith G Hemani G. Mendelian randomization: genetic anchors for causal inference in epidemiological studies. Hum Mol Genet. (2014) 23:R89–98. 10.1093/hmg/ddu328 25064373 PMC4170722

[18] Birney E. Mendelian randomization. Cold Spring Harb Perspect Med. (2022) 12:4. 10.1101/cshperspect.a041302 PMC9121891 34872952

[19] Emdin CA Khera AV Kathiresan S. Mendelian randomization. JAMA. (2017) 318:1925–6. 10.1001/jama.2017.17219 29164242

[20] Skrivankova VW Richmond RC Woolf BAR Yarmolinsky J Davies NM Swanson SA. Strengthening the reporting of observational studies in epidemiology using mendelian randomization: the STROBE-MR statement. JAMA. (2021) 326:1614–21. 10.1001/jama.2021.18236 34698778

[21] Initiative C-HG. Mapping the human genetic architecture of COVID-19. Nature. (2021) 600:472–7. 10.1038/s41586-021-03767-x 34237774 PMC8674144

[22] Corvin A Sullivan PF. What next in schizophrenia genetics for the psychiatric genomics consortium? Schizophr Bull. (2016) 42:538–41. 10.1093/schbul/sbw014 26994396 PMC4838114

[23] Howard DM Adams MJ Clarke TK Hafferty JD Gibson J Shirali M. Genome-wide meta-analysis of depression identifies 102 independent variants and highlights the importance of the prefrontal brain regions. Nat Neurosci. (2019) 22:343–52. 10.1038/s41593-018-0326-7 30718901 PMC6522363

[24] Strausz S Ruotsalainen S Ollila HM Karjalainen J Kiiskinen T Reeve M. Genetic analysis of obstructive sleep apnoea discovers a strong association with cardiometabolic health. Eur Respir J. (2021) 57:5. 10.1183/13993003.03091-2020 33243845

[25] Burgess S Thompson SG. Bias in causal estimates from Mendelian randomization studies with weak instruments. Stat Med. (2011) 30:1312–23. 10.1002/sim.4197 21432888

[26] Burgess S Thompson SG Collaboration CCG. Avoiding bias from weak instruments in Mendelian randomization studies. Int J Epidemiol. (2011) 40:755–64. 10.1093/ije/dyr036 21414999

[27] Zhang J Lu H Zeng H Zhang S Du Q Jiang T. The differential psychological distress of populations affected by the COVID-19 pandemic. Brain Behav Immun. (2020) 87:49–50. 10.1016/j.bbi.2020.04.031 32304883 PMC7156946

[28] Niedzwiedz CL Benzeval M Hainey K Leyland AH Katikireddi SV. Psychological distress among people with probable COVID-19 infection: analysis of the UK household longitudinal study. BJPsych Open. (2021) 7:e104. 10.1192/bjo.2021.63 34001295 PMC8134894

[29] Chen F Zheng D Liu J Gong Y Guan Z Lou D. Depression anxiety among adolescents during COVID-19: a cross-sectional study. Brain Behav Immun. (2020) 88:36–8. 10.1016/j.bbi.2020.05.061 32464156 PMC7247496

[30] Renaud-Charest O Lui LMW Eskander S Ceban F Ho R Di Vincenzo JD. Onset and frequency of depression in post-COVID-19 syndrome: a systematic review. J Psychiatr Res. (2021) 144:129–37. 10.1016/j.jpsychires.2021.09.054 34619491 PMC8482840

[31] Magnusdottir I Lovik A Unnarsdottir AB McCartney D Ask H Koiv K. Acute COVID-19 severity and mental health morbidity trajectories in patient populations of six nations: an observational study. Lancet Public Health. (2022) 7:e406–16. 10.1016/S2468-2667(22)00042-1 35298894 PMC8920517

[32] Li Y Scherer N Felix L Kuper H. Prevalence of depression, anxiety and post-traumatic stress disorder in health care workers during the COVID-19 pandemic: a systematic review and meta-analysis. PLoS ONE. (2021) 16:e0246454. 10.1371/journal.pone.0246454 33690641 PMC7946321

[33] Brooks SK Webster RK Smith LE Woodland L Wessely S Greenberg N. The psychological impact of quarantine and how to reduce it: rapid review of the evidence. Lancet. (2020) 395:912–20. 10.1016/S0140-6736(20)30460-8 32112714 PMC7158942

[34] Maehlisen MH Pasgaard AA Mortensen RN Vardinghus-Nielsen H TorpPedersen CH Boggild H. Perceived stress as a risk factor of unemployment: a register-based cohort study. BMC Public Health. (2018) 18:728. 10.1186/s12889-018-5618-z 29895286 PMC5998595

[35] Wang C Pan R Wan X Tan Y Xu L Ho CS. Immediate psychological responses and associated factors during the initial stage of the 2019 coronavirus disease (COVID-19) epidemic among the general population in China. Int J Environ Res Public Health. (2020) 17:5. 10.3390/ijerph17051729 32155789 PMC7084952

[36] Xiang YT Yang Y Li W Zhang L Zhang Q Cheung T. Timely mental health care for the 2019 novel coronavirus outbreak is urgently needed. Lancet Psychiatry. (2020) 7:228–9. 10.1016/S2215-0366(20)30046-8 32032543 PMC7128153

[37] Taylor S Asmundson GJG. Life in a post-pandemic world: What to expect of anxiety-related conditions and their treatment. J Anxiety Disord. (2020) 72:102231. 10.1016/j.janxdis.2020.102231 32447204 PMC7252157

[38] Schou TM Joca S Wegener G Bay-Richter C. Psychiatric and neuropsychiatric sequelae of COVID-19-A systematic review. Brain Behav Immun.(2021) 97:328–48. 10.1016/j.bbi.2021.07.018 34339806 PMC8363196

[39] Salvagioni DAJ Melanda FN Mesas AE Gonzalez AD Gabani FL Andrade SM. Physical, psychological and occupational consequences of job burnout: a systematic review of prospective studies. PLoS ONE. (2017) 12:e0185781. 10.1371/journal.pone.0185781 28977041 PMC5627926

[40] Morin CM Carrier J. The acute effects of the COVID-19 pandemic on insomnia and psychological symptoms. Sleep Med. (2021) 77:346–7. 10.1016/j.sleep.2020.06.005 32595107 PMC7274952

[41] Mirchandaney R Barete R Asarnow LD. Moderators of cognitive behavioral treatment for insomnia on depression and anxiety outcomes. Curr Psychiatry Rep. (2022) 24:121–8. 10.1007/s11920-022-01326-3 35061137 PMC8948126

[42] Cacioppo JT Cacioppo S Capitanio JP Cole SW. The neuroendocrinology of social isolation. Annu Rev Psychol. (2015) 66:733–67. 10.1146/annurev-psych-010814-015240 25148851 PMC5130104

[43] Vgontzas AN Fernandez-Mendoza J Lenker KP Basta M Bixler EO Chrousos GP. Hypothalamic-pituitary-adrenal (HPA) axis response to exogenous corticotropin-releasing hormone (CRH) is attenuated in men with chronic insomnia. J Sleep Res. (2022) 31:e13526. 10.1111/jsr.13526 34825417

[44] Morin CM Bjorvatn B Chung F Holzinger B Partinen M Penzel T. Insomnia, anxiety, and depression during the COVID-19 pandemic: an international collaborative study. Sleep Med. (2021) 87:38–45. 10.1016/j.sleep.2021.07.035 34508986 PMC8425785

[45] Miller AH Raison CL. The role of inflammation in depression: from evolutionary imperative to modern treatment target. Nat Rev Immunol. (2016) 16:22–34. 10.1038/nri.2015.5 26711676 PMC5542678

[46] Bohmwald K Galvez NMS Rios M Kalergis AM. Neurologic alterations due to respiratory virus infections. Front Cell Neurosci. (2018) 12:386. 10.3389/fncel.2018.00386 30416428 PMC6212673

[47] Dantzer R. Neuroimmune interactions: from the brain to the immune system and vice versa. Physiol Rev. (2018) 98:477–504. 10.1152/physrev.00039.2016 29351513 PMC5866360

[48] Mazza MG De Lorenzo R Conte C Poletti S Vai B Bollettini I. Anxiety and depression in COVID-19 survivors: Role of inflammatory and clinical predictors. Brain Behav Immun. (2020) 89:594–600. 10.1016/j.bbi.2020.07.037 32738287 PMC7390748

[49] Wu Y Xu X Chen Z Duan J Hashimoto K Yang L. Nervous system involvement after infection with COVID-19 and other coronaviruses. Brain Behav Immun. (2020) 87:18–22. 10.1016/j.bbi.2020.03.031 32240762 PMC7146689

[50] Jones DP Wootton RE Gill D Carter AR Gunnell D Munafo MR. Mental health as a mediator of the association between educational inequality and cardiovascular disease: a mendelian randomization study. J Am Heart Assoc. (2021) 10:e019340. 10.1161/JAHA.120.019340 34472355 PMC8649303

[51] Zeng Y Cao S Yang H. The causal role of gastroesophageal reflux disease in anxiety disorders and depression: a bidirectional Mendelian randomization study. Front Psychiatry. (2023) 14:1135923. 10.3389/fpsyt.2023.1135923 36911112 PMC9992201

[52] Wang Q Xu R Volkow ND. Increased risk of COVID-19 infection and mortality in people with mental disorders: analysis from electronic health records in the United States. World Psychiatry. (2021) 20:124–30. 10.1002/wps.20806 33026219 PMC7675495

[53] Verdolini N Amoretti S Montejo L Garcia-Rizo C Hogg B Mezquida G. Resilience and mental health during the COVID-19 pandemic. J Affect Disord. (2021) 283:156–64. 10.1016/j.jad.2021.01.055 33556749 PMC7845537

[54] Pal R Banerjee M Bhadada SK. Cortisol concentrations and mortality from COVID-19. Lancet Diabetes Endocrinol. (2020) 8:809. 10.1016/S2213-8587(20)30304-1 PMC7491987 32946817

[55] Popescu M Terzea DC Carsote M Ghenea AE Costache A Popescu IAS. COVID-19 infection: from stress-related cortisol levels to adrenal glands infarction. Rom J Morphol Embryol. (2022) 63:39–48. 10.47162/RJME.63.1.03 36074666 PMC9593124

[56] Stone LB McCormack CC Bylsma LM. Cross system autonomic balance and regulation: Associations with depression and anxiety symptoms. Psychophysiology. (2020) 57:e13636. 10.1111/psyp.13636 33460174 PMC8054991

[57] Gosain R Gage-Bouchard E Ambrosone C Repasky E Gandhi S. Stress reduction strategies in breast cancer: review of pharmacologic and non-pharmacologic based strategies. Semin Immunopathol. (2020) 42:719–34. 10.1007/s00281-020-00815-y 32948909 PMC7704484

[58] Sun N Qin YJ Xu C Xia T Du ZW Zheng LP. Design of fast-onset antidepressant by dissociating SERT from nNOS in the DRN. Science. (2022) 378:390–8. 10.1126/science.abo3566 36302033

[59] Xing X Hu X. Risk factors of cytokine release syndrome: stress, catecholamines, and beyond. Trends Immunol. (2023) 44:93–100. 10.1016/j.it.2022.12.003 36586780

[60] Wang S Quan L Chavarro JE Slopen N Kubzansky LD Koenen KC. Associations of depression, anxiety, worry, perceived stress, and loneliness prior to infection with risk of post-COVID-19 conditions. JAMA Psychiatry. (2022) 79:1081–91. 10.1001/jamapsychiatry.2022.2640 36069885 PMC9453634

